# Aldosterone-producing adenoma is associated with urolithiasis in primary aldosteronism

**DOI:** 10.1530/EC-23-0056

**Published:** 2023-08-11

**Authors:** Victor Jing-Wei Kang, Bo-Ching Lee, Jia-Zheng Huang, Vin-Cent Wu, Yen-Hung Lin, Chin-Chen Chang, TAIPAI group

**Affiliations:** 1 Departments of Medical Imaging, National Taiwan University Hospital, Taipei, Taiwan; 2 Departments of Internal Medicine, National Taiwan University Hospital, Taipei, Taiwan

**Keywords:** hypertension, humans, primary aldosteronism, renal stone

## Abstract

Primary aldosteronism (PA) is associated with urolithiasis as it causes hypercalciuria and hypocitraturia. However, the influence of different subtypes of PA on urinary stone formation remains unclear. This study aimed to evaluate the association between aldosterone-producing adenoma (APA) and the burden of urolithiasis in patients with PA. In the present study, we enrolled 312 patients with PA from a prospectively maintained database, of whom 179 had APA. Clinical, biochemical, and imaging data (including the presence, volume, and density of urinary stones on abdominal computed tomography) were compared between groups, with employment of propensity score matching (PSM) analysis to balance possible confounding factors. Kaplan–Meier analysis was used to estimate the acute renal colic event during follow-up. After PSM for age, sex, serum calcium, phosphate, blood urea nitrogen, creatinine, and uric acid, the APA and non-APA groups had 106 patients each. Patients with APA had higher serum intact parathyroid hormone (iPTH) (79.1 ± 45.0 vs 56.1 ± 30.3, *P* < 0.001) and a higher prevalence of urolithiasis (27.4% vs 12.3%, *P* = 0.006) than non-APA patients. During follow-up, a higher incidence of acute renal colic events was noted in the APA group than the non-APA group (*P* = 0.011); this association remained significant (*P* = 0.038) after adjustment for age and sex in Cox-regression analysis. Our data suggest that APA is associated with a heavier burden of urolithiasis and higher incidence of renal colic events compared to the non-APA subtype of PA.

## Introduction

Primary aldosteronism (PA) is the most common cause of secondary hypertension; approximately 5–10% of hypertensive patients may have PA (1). However, the incidence of PA may be as high as 20% in patients with treatment-resistant hypertension (2). Aldosterone-producing adenoma (APA) and idiopathic hyperaldosteronism (IHA) are the two main subtypes of PA (3). While APA can be cured with unilateral adrenalectomy, IHA is usually managed with pharmacological approaches such as mineralocorticoid receptor antagonists (4). Patients with APA tend to have higher levels of aldosterone secretion and more severe hypertension than patients with IHA (5, 6).

Excessive aldosterone is associated with secondary hyperparathyroidism, osteoporosis, calcium metabolic disorder, hypercalciuria (7, 8, 9, 10), and acidic urine (11) and can promote the development of kidney stone disease. Clinically, patients with PA are reported to have a higher incidence of urolithiasis and larger kidney stones than patients with essential hypertension (12). However, the contribution of the different subtypes of PA to kidney stone disease remains poorly understood.

Adrenal computed tomography (CT) is an essential component of the diagnosis of PA and is used to localize adrenal nodules, to rule out adrenal malignancy, and to guide subsequent adrenal venous sampling (AVS) (13, 14, 15). Thus, the burden of kidney stone disease in patients with various subtypes of PA can be quantitatively evaluated utilizing CT images. The present study aimed to investigate the influence of different subtypes of PA on urolithiasis, and we employed propensity score matching (PSM) analysis to balance possible confounding factors.

## Materials and methods

### Patient enrollment

We enrolled 312 patients with PA from a prospectively maintained database at National Taiwan University Hospital (NTUH) between September 2006 and March 2019. A total of 179 patients were diagnosed with APA and 133 patients were diagnosed with IHA. This study was approved by the institutional review board of NTUH, and the requirement for informed consent was waived. Data on clinical parameters – including demographic data, follow-up data, laboratory data, and adrenal CT – were recorded.

### Laboratory measurements

Plasma aldosterone concentration (PAC) and plasma renin activity (PRA) were measured using specific radioimmunoassay kits (Aldosterone Maia Kit; Adaltis Italia, Bologna, Italy and DiaSorin, Stillwater, MN, USA, respectively). The aldosterone-to-renin ratio (ARR) was calculated as PAC/PRA.

### Diagnostic criteria for APA

The screening, confirmation, and subtype identification of PA were conducted following the standard Taiwan Primary Aldosteronism Investigators (TAIPAI) protocol and the consensus on aldosteronism in Taiwan (16). The diagnosis of APA was established based on the modified Four Corners criteria, which include (1) excess aldosterone production, as defined by an ARR > 35, TAIPAI score > 60% (17), and seated post-**–**saline loading PAC > 16 ng/dL or urine aldosterone > 12 mg/24 h; (2) identification of adrenal nodules on CT; (3) lateralization of aldosterone hypersecretion based on AVS or dexamethasone suppression NP-59 single-photon emission CT; and (4) pathological evidence of adenoma after adrenalectomy and subsequent clinical improvement defined as either complete resolution of hypertension or partial resolution of hypertension, potassium, PAC, and PRA (18).

### Imaging analysis

Adrenal CT images were available for all patients. Two radiologists (V J K and B C L, with 3 and 9 years of experience, respectively) independently evaluated the CT images on a standard imaging workstation while blinded to the PA subtypes. Imaging parameters of kidney stone disease on adrenal CT were recorded, including the location, laterality, diameter, volume, and Hounsfield units (HUs) of the identified stones.

### Statistical analysis

All statistical analyses were performed using SPSS version 26 (IBM). Categorical variables were analyzed using Fisher’s exact test or the chi-square test, as appropriate. The independent two-sample *t*-test was employed to compare continuous variables. Continuous variables with skewed distributions, including PAC and ARR, were analyzed using the Mann–Whitney *U* test. PSM was used to balance possible confounders between patients with APA and patients with IHA. Clinical variables including age, sex, serum calcium, phosphate, blood urea nitrogen, creatinine, and uric acid were included in the PSM analysis. The cumulative rates of acute renal colic were estimated from follow-up data using Kaplan–Meier analysis and the log-rank test. Multivariable Cox regression analysis was used to adjust for age and sex. The survival time was defined as the interval between the date of adrenal CT and a clinical episode of acute renal colic or the date of last follow-up. Patients who died or were lost to follow-up were censored at the date of their last outpatient clinic visit.

## Results

Of the 312 patients in this study, 179 were diagnosed with APA and 133 were diagnosed with IHA. The demographic features of the two groups are compared in Table 1. Before PSM, the patients with APA had lower serum levels of potassium, calcium, and phosphate, had higher serum levels of PAC, ARR, creatinine, 24-h urine calcium and intact parathyroid hormone (iPTH), more frequently used hypertensive medication, and more frequently had renal or ureteral stones (47, 26.3% vs 16, 12.0%; *P* = 0.002) than patients with IHA. The other imaging characteristics of kidney stone disease, including the lateralization, diameter, volume, and HUs of the stones, were not significantly different between groups (Table 2).

**Table 1:** Demographic characteristics of the patients with APA and patients with IHA before and after propensity score matching.

	Before propensity score matching			After propensity score matching		
	APA (*n* = 179)	IHA (*n* = 133)	*P*-value	APA (*n* = 106)	IHA (*n* = 106)	*P* value
Age, years	51.2 ± 10.9	53.2 ± 11.8	0.122	50.9 ± 11.3	53.6 ± 11.9	0.097
Sex, male	85 (47.5%)	55 (41.4%)	0.281	52 (49.1%)	45 (42.5%)	0.336
BMI, kg/m^2^	24.8 ± 4.2	25.5 ± 3.9	0.119	24.9 ± 3.9	25.8 ± 3.9	0.110
SBP, mmHg	153.5 ± 20.1	150.7 ± 19.5	0.217	155.7 ± 20.9	150.4 ± 20.0	0.060
DBP, mmHg	92.9 ± 14.1	90.2 ± 13.2	0.085	93.5 ± 14.4	89.8 ± 13.5	0.055
HTN drugs, *n*	2.2 ± 1.2	1.9 ± 1.3	0.019^a^	2.2 ± 1.3	1.9 ± 1.4	0.062
PAC, ng/dL	58.1 ± 39.0	48.4 ± 31.8	0.016^a^	55.0 ± 31.8	48.5 ± 32.8	0.070
PRA, ng/mL/h	0.6 ± 2.2	0.6 ± 0.9	0.719	0.8 ± 2.8	0.5 ± 0.8	0.118
ARR	1225.7 ± 2562.1	329.3 ± 516.2	<0.001^b^	1042.6 ± 2182.6	327.1 ± 436.1	0.026^a^
Log PAC	1.7 ± 0.3	1.6 ± 0.3	0.039^a^	1.7 ± 0.3	1.6 ± 0.3	0.070
Log PRA	–0.7 ± 0.7	–0.6 ± 0.5	0.010^a^	−0.8 ± 0.7	−0.6 ± 0.5	0.118
Log ARR	2.4 ± 0.8	2.2 ± 0.6	0.001^c^	2.4 ± 0.7	2.2 ± 0.6	0.026^a^
Na, mmol/L	141.0 ± 2.7	140.8 ± 2.8	0.538	141.2 ± 2.9	140.8 ± 2.9	0.840
K, mmol/L	3.5 ± 0.6	3.8 ± 0.5	<0.001^b^	3.5 ± 0.6	3.8 ± 0.5	0.002^c^
Ca, mmol/L	2.3 ± 0.1	2.4 ± 0.3	0.001^c^	2.3 ± 0.2	2.4 ± 0.3	<0.001^b^
P, mmol/L	3.3 ± 0.6	3.5 ± 0.6	0.009^c^	3.3 ± 0.6	3.5 ± 0.6	0.010^a^
Cre, mg/dL	0.9 ± 0.5	0.8 ± 0.3	0.011^a^	0.9 ± 0.4	0.8 ± 0.2	0.039^a^
BUN, mg/dL	14.6 ± 5.9	14.8 ± 5.4	0.724	14.6 ± 6.2	14.8 ± 5.0	0.477
eGFR, mL/min/1.73 m^2^	95.2 ± 26.3	95.7 ± 24.7	0.749	99.1 ± 28.3	96.4 ± 24.6	0.630
UA, mg/dL	5.7 ± 1.6	5.7 ± 1.8	0.860	5.7 ± 1.6	5.7 ± 1.8	0.398
iPTH, pg/mL	77.0 ± 42.3	57.7 ± 31.1	<0.001^b^	79.1 ± 45.0	56.1 ± 30.3	<0.001^b^
Urine Ca (24 h), mmol/L	2.6 ± 1.2	2.1 ± 1.2	0.004^c^	2.6 ± 1.3	2.1 ± 1.3	0.012^a^

Age, sex, serum calcium, serum phosphate, blood urea nitrogen, creatinine, and uric acid were matched in propensity score analysis.

^a^*P* < 0.05; ^b^*P* < 0.001; ^c^*P* < 0.01.

ARR, aldosterone-to-renin ratio; BMI, body mass index; BUN, blood urea nitrogen; Ca, serum calcium; Cre, creatinine; DBP, diastolic blood pressure; eGFR, estimated glomerular filtration rate; HTN, hypertension; iPTH, intact parathyroid hormone; K, serum potassium; Na, serum sodium; P, serum phosphate; PAC, plasma aldosterone concentration; PRA, plasma renin activity; SBP, systolic blood pressure; UA, uric acid.

**Table 2:** Characteristics of kidney stone disease in patients with APA and patients with IHA before and after propensity score matching.

	Before propensity score matching			After propensity score matching		
	APA (*n* = 179)	IHA (*n* = 133)	*P* value	APA (*n* = 106)	IHA (*n* = 106)	*P* value
Presence of renal or ureteral stones	47 (26.3%)	16 (12.0%)	0.002^a^	29 (27.4%)	13 (12.3%)	0.006^a^
Unilateral/bilateral	30/17	11/5	0.772	18/11	10/3	0.485
Maximal diameter, mm	5.0 ± 3.2	4.3 ± 1.5	0.693	5.4 ± 3.7	4.4 ± 1.6	0.462
Total stone volume, mm^3^	206.6 ± 356.5	186.3 ± 308.6	0.872	259.1 ± 377.9	199.4 ± 342.7	0.363
Hounsfield unit	473.9 ± 332.7	497.8 ± 325.0	0.607	536.0 ± 362.5	429.9 ± 274.4	0.523

Age, sex, serum calcium, serum phosphate, blood urea nitrogen, creatinine, and uric acid were matched in propensity score analysis.

^a^*P* < 0.01.

APA, aldosterone-producing adenoma; IHA, idiopathic hyperaldosteronism.

After PSM for age, sex, serum calcium, phosphate, blood urea nitrogen, creatinine, and uric acid, 106 patients remained in each group. Both groups were similar in terms of age, sex, body mass index, blood pressure, and hypertensive medications. The matched patients with APA had lower serum levels of potassium, calcium, and phosphate, higher serum levels of ARR, 24 h urine calcium, iPTH, and creatinine, and a higher frequency of renal or ureteral stones (29, 27.4% vs 13, 12.3%; *P* = 0.006) than patients with IHA (Table 2). The other imaging characteristics of kidney stone disease were similar between the PSM groups.

**Figure 1:**
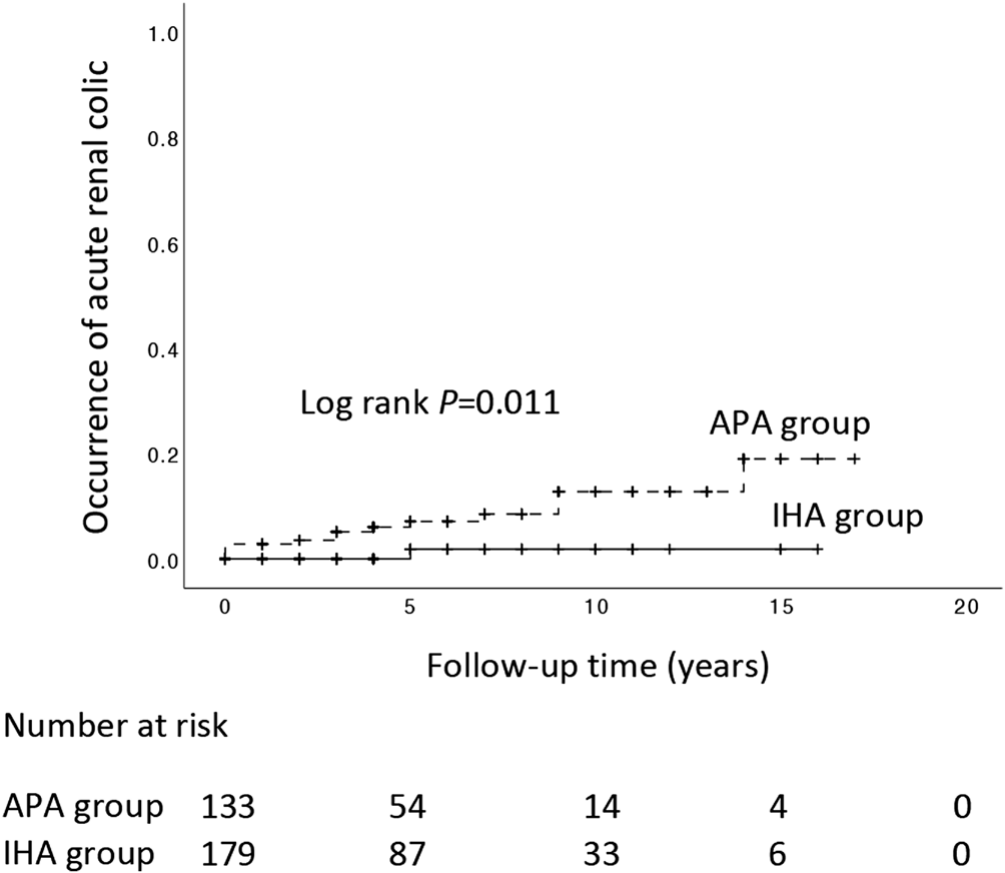
The Kaplan–Meier analysis showed that APA was associated with a significantly higher incidence of acute renal colic compared to IHA (*P* = 0.011).

Next, we examined the association of the two subtypes of PA with acute renal colic using follow-up data. During the follow-up period of 5.1 ± 4.1 years, patients with APA developed 14 recorded episodes of acute renal colic compared to 1 recorded episode of acute renal colic among the patients with IHA. Kaplan–Meier analysis showed that APA was associated with a significantly higher incidence of acute renal colic compared to IHA (*P* = 0.011; Fig. 1). In multivariable Cox regression analysis to adjust for age and sex, APA remained associated with a significantly higher risk of acute renal colic compared to IHA (95% confidence interval: 1.1‒65.6, *P* = 0.038).

## Discussion

This study indicates that patients with APA have a higher prevalence of kidney stone disease than patients with IHA; this association remained significant after PSM for potential confounders. APA was also associated with a higher incidence of acute renal colic events than IHA during follow-up, which suggests that the risk of kidney stone disease in APA may be underappreciated among patients with PA.

Kabadi *et al.* provided the first case report of recurrent renal calculi as a major manifestation of PA (10). High plasma levels of aldosterone lead to volume expansion and retention of sodium and water, which decreases the absorption of sodium and calcium in the proximal tubules and results in hypercalciuria (19, 20). In addition, high plasma aldosterone decreases the plasma level of potassium, which influences reabsorption of phosphate. Decreased serum phosphate leads to absorption of intestinal calcium, bone resorption, and calcitriol synthesis, which causes hypercalciuria (21, 22). Moreover, hyperaldosteronism causes intracellular acidosis, which increases the absorption of citrate in the proximal tubules and induces hypocitraturia (9, 23). A global epidemiological study showed that the prevalence of kidney stone disease ranges from 1.7% to 14.8% (24), while the prevalence of kidney stone disease in patients with PA is approximately 24% (12). Though this evidence suggested that PA may play a significant role in the formation of urolithiasis, the relationship between the subtypes of PA and urolithiasis remained poorly understood.

In the present study, we found that patients with APA have a higher burden of kidney stone disease and elevated serum levels of iPTH than patients with IHA. iPTH is synthesized by one or more of the four parathyroid glands and plays an important role in regulating the circulating levels of calcium and phosphate through its action on the bones, kidneys, and intestine. PA was associated with a higher incidence of hyperparathyroidism, higher level of iPTH, lower level of vitamin D, hypercalciuria, and a higher incidence of osteopenia/osteoporosis compared to patients with essential hypertension (25, 26) and can be reversed by mineralocorticoid receptor antagonists (e.g. spironolactone) or adrenalectomy (27). Rossi *et al.* demonstrated that elevated iPTH levels are characteristic of APA and can serve as a valuable marker for subtyping PA patients who may benefit from additional management (28). The APA subtype of PA may be associated with more severe aldosteronism than the IHA subtype, as evidenced by lower serum potassium and higher ARR levels. This finding explains the higher levels of iPTH observed in patients with APA in this study and, in turn, indicates how iPTH may result in urolithiasis. However, further study is required to identify the mechanisms that explain this relationship.

There are several limitations to our present study. First, there were significant differences in the clinical characteristics and laboratory data of the patients with the APA and IHA subtypes of PA; though we used PSM to balance these clinical confounders, some bias may still remain. Secondly, a variety of lifestyle and medication risk factors related to urolithiasis, such as diet, fluid intake, smoking, type 2 diabetes mellitus, and use of thiazide (29, 30, 31), were not analyzed in this study. Thirdly, we did not use dual-energy CT in this study; thus, further studies are warranted to elucidate and compare the composition of the renal stones in each subtype of PA.

In conclusion, this study provides evidence that patients with APA have a higher burden of kidney stone disease and higher incidence of acute renal colic compared to patients with IHA, which may be the result of the higher serum iPTH related to APA. These findings highlight the importance of appropriate treatment such as adrenalectomy and regular screening for kidney stone disease in this specific subgroup of patients with PA.

## Declaration of interest

None declared.

## Funding

This work was supported by grants from the National Taiwan University http://dx.doi.org/10.13039/501100006477 Hospital (Lee BC, 111-X0002 & 112-S019) and National Science and Technology Councilhttp://dx.doi.org/10.13039/501100020950 of Taiwan (111-2314-B-002-250-MY2).

## Author contribution statement

Victor Jing-Wei Kang **–** data collection, imaging analysis, write-up; Bo-Ching Lee **–** project concept and design, data collection, imaging analysis, data analysis, write-up; Chin-Chen Chang **–** project concept and design; Jia-Zheng Huang **–** data collection, imaging analysis; Vin-Cent Wu project concept and design, data collection, critical revisions; Yen-Hung Lin **–** project concept and design, data collection, imaging analysis, critical revisions.
